# Congenital Occlusion of the Posterior Nares

**Published:** 1887-02

**Authors:** Alvin A. Hubbell


### CONGENITAL OCCLUSION OF THE POSTERIOR NARES.

*Editor Buffalo Medical and Surgical Journal:*

DEAR SIR—In my paper on "Congenital Occlusion of the Posterior Nares," published in the December number of the JOURNAL, I stated that I was unable to verify a case of Gosselin, referred to by Dr. Geo. M. Lefferts, of New York, in the "*International Encyclopædia of Surgery*," and therefore did not include it in my report. Dr. Lefferts has since very kindly informed me that the date in the reference in the *Surgery* was printed "1877," whereas it should have been "1876." The original report of the case may be found, therefore, in the "*Gazette Medicale de Paris*," at page 430, of number 36, September 2, 1876.

This case was a girl seventeen or eighteen years of age, with "unilateral obstruction by the posterior nasal fossa," who had had symptoms of impaired respiration, "felt by the young patient as if she had been suffering from a coryza." Attempts were made in vain to pass sounds, or to inject water through the affected side into the pharynx. By carrying the finger behind the velum palti to the point where the posterior opening should have been, Gosselin became "satisfied of the presence of a resisting plane," and he concluded that "there was an obliteration similar to the one which M. Bitot had spoken of." No operation was performed, although he thought one justifiable. He does not state on which side the obstruction existed, nor does he inform us whether it was bony or membranous, but I infer that it was bony.

This gives me a total of eighteen reported cases, and adds one more to the number of cases of complete bony (?) occlusion, limited to one choana.

Yours truly,

ALVIN A. HUBBELL, M. D.

